# Dysfunction of the Heteromeric K_V_7.3/K_V_7.5 Potassium Channel is Associated with Autism Spectrum Disorders

**DOI:** 10.3389/fgene.2013.00054

**Published:** 2013-04-16

**Authors:** Mette Gilling, Hanne B. Rasmussen, Kirstine Calloe, Ana F. Sequeira, Marta Baretto, Guiomar Oliveira, Joana Almeida, Marlene B. Lauritsen, Reinhard Ullmann, Susanne E. Boonen, Karen Brondum-Nielsen, Vera M. Kalscheuer, Zeynep Tümer, Astrid M. Vicente, Nicole Schmitt, Niels Tommerup

**Affiliations:** ^1^Wilhelm Johannsen Centre for Functional Genome Research, Department of Cellular and Molecular Medicine, University of CopenhagenCopenhagen, Denmark; ^2^Section for Neurogenetics, Department of Cellular and Molecular Medicine, University of CopenhagenCopenhagen, Denmark; ^3^Danish National Research Foundation Centre for Cardiac Arrhythmia, Department of Biomedical Sciences, University of CopenhagenCopenhagen, Denmark; ^4^Instituto Gulbenkian de CiênciaOeiras, Portugal; ^5^Instituto Nacional de Saúde Dr. Ricardo JorgeLisbon, Portugal; ^6^Centro de Desencolcimento da Criança, Centro Investigação e Formação clinica, Hospital Pediátrico, Centro Hospitalar e Universitário de Coimbra, Faculty of Medicine, University of CoimbraCoimbra, Portugal; ^7^Regional Centre for Child and Adolescent Psychiatry, Aarhus University HospitalAarhus, Denmark; ^8^Max-Planck Institute for Molecular GeneticsBerlin, Germany; ^9^Kennedy Center, Copenhagen University HospitalRigshospitalet, Glostrup, Denmark

**Keywords:** Autism, *KCNQ3*, *KCNQ5*, K_V_7.3, K_V_7.5, translocation, SNP

## Abstract

Heterozygous mutations in the *KCNQ3* gene on chromosome 8q24 encoding the voltage-gated potassium channel K_V_7.3 subunit have previously been associated with rolandic epilepsy and idiopathic generalized epilepsy (IGE) including benign neonatal convulsions. We identified a *de novo* *t*(3;8) (q21;q24) translocation truncating *KCNQ3* in a boy with childhood autism. In addition, we identified a c.1720C > T [p.P574S] nucleotide change in three unrelated individuals with childhood autism and no history of convulsions. This nucleotide change was previously reported in patients with rolandic epilepsy or IGE and has now been annotated as a very rare SNP (rs74582884) in dbSNP. The p.P574S K_V_7.3 variant significantly reduced potassium current amplitude in *Xenopus laevis* oocytes when co-expressed with K_V_7.5 but not with K_V_7.2 or K_V_7.4. The nucleotide change did not affect trafficking of heteromeric mutant K_V_7.3/2, K_V_7.3/4, or K_V_7.3/5 channels in HEK 293 cells or primary rat hippocampal neurons. Our results suggest that dysfunction of the heteromeric K_V_7.3/5 channel is implicated in the pathogenesis of some forms of autism spectrum disorders, epilepsy, and possibly other psychiatric disorders and therefore, *KCNQ3* and *KCNQ5* are suggested as candidate genes for these disorders.

## Introduction

Autism spectrum disorders (ASD, OMIM 209850) are neurodevelopmental disorders with early childhood onset and a lifelong persistence. They are characterized by severe impairments in reciprocal social interaction and communication as well as by stereotypic behavior or interests. The prevalence of ASDs is estimated to be 9 in 1000 (Autism and Developmental Disabilities Monitoring Network, 2009; Baron-Cohen et al., 2009) with a male to female ratio of 4:1 (Folstein and Rosen-Sheidley, 2001). Intellectual disability (ID) occurs in approximately 50% of ASD individuals; approximately 20–30% have comorbid epilepsy (State, 2010; Tuchman et al., 2010; Kohane et al., 2012) Furthermore, 72% of children diagnosed with ASD have at least one additional psychiatric disorder such as attention deficit hyperactivity disorder (ADHD), major depression, schizophrenia, phobia, obsessive compulsive disorder (OCD) (Stahlberg et al., 2004; Leyfer et al., 2006; Abdallah et al., 2011). Common genetic etiologies have been identified for ASD, ID, epilepsy and various psychiatric disorders which emphasizes the likely overlap in pathogenesis for these disorders (Moreno-De-Luca et al., 2010; Gregor et al., 2011; Duong et al., 2012). Twin and family studies show that genetic factors are of profound importance for the development of ASD (Folstein and Rutter, 1977; Bailey et al., 1995), but only few genes have been accepted as ASD susceptibility genes. Likely explanations are the apparently high degree of locus heterogeneity rendering it difficult to identify mutations in a gene in a convincing number of patients, the pleiotropic effects of many neuronal disease genes making the connection between genotype and phenotype less obvious (Duong et al., 2012; Iossifov et al., 2012; O’Roak et al., 2012), and the likely substantial contribution of *de novo* mutations (Sebat, 2007; Levy et al., 2011; Iossifov et al., 2012; Neale et al., 2012). In addition, point mutations (Jamain et al., 2003; Feng et al., 2006; Berkel et al., 2010; O’Roak et al., 2012), rare genomic copy number variants (CNVs), and recurrent CNVs (Ullmann et al., 2007; de Kovel et al., 2010; Moreno-De-Luca et al., 2010) that increase the risk of ASD and/or epilepsy, ID, and psychiatric disorders may be transmitted from apparently normal parents.

Mutations in the genes *KCNQ2* and *KCNQ3* cause idiopathic generalized epilepsy (IGE) (Neubauer et al., 2008). These include benign neonatal epilepsy (Biervert et al., 1998; Charlier et al., 1998) as well as benign childhood epilepsy with centrotemporal spikes (rolandic epilepsy) (Neubauer et al., 2008) consistent with dysregulation of neuronal excitability. Intriguingly, >20% of patients with rolandic epilepsy have cognitive deficits and >10% have behavioral problems (ADHD, anxiety, depression, and pervasive developmental disorder (PDD) (Tovia et al., 2011). Furthermore, 40% of patients with benign familial neonatal convulsions show delayed psychomotor development or ID (Steinlein et al., 2007) and >25% of patients with IGE have comorbid mental disorders (Akanuma et al., 2008). *KCNQ3* is one of five *KCNQ* genes (*KCNQ1-5*) encoding the K_V_7 family of voltage-gated potassium channels (Brown and Passmore, 2009). Four of these genes (*KCNQ2-5*) are expressed in the central nervous system both on RNA and protein level (Brown and Passmore, 2009) and are therefore excellent candidate susceptibility genes for a wide range of neuronal disorders. K_V_7.3 forms heterotetrameric channels with K_V_7.2 (Schroeder et al., 1998), K_V_7.4 (Kubisch et al., 1999), and K_V_7.5 (Schroeder et al., 2000). K_V_7.2/K_V_7.3 heteromeric channels primarily localize at the axon initial segment (AIS) and underlie the M-current involved in regulation of neuronal excitability (Wang et al., 1998; Schroeder et al., 2000).

In this study we have investigated *KCNQ3* gene variability in two independent ASD cohorts from Portugal and Denmark.

## Materials and Methods

### Clinical information, patient A

Patient A is a Danish boy who carries a *de novo* balanced translocation *t*(3;8) (q21;q24). He was born in 1998 as the second child of non-consanguineous, Caucasian, healthy parents aged 37 (mother) and 38 (father) at the time of birth. Both parents have academic degrees. According to the parents the older sister is both intellectually and socially very well-functioning. The pregnancy was normal and the delivery at gestational age 40 + 2 was uncomplicated with Apgar scores 9/1 and 10/5. The birth weight was 3900 g; birth length 53 cm; and head circumference 36 cm. Growth parameters are currently still normal. No dysmorphic features were noted at birth and hearing was normal.

In the neonatal period the parents noticed an abnormal social interaction with him. Later on it was apparent that both verbal and social development was delayed; however motor milestones were achieved normally. Genetic testing for the fragile X syndrome was negative and metabolic screening showed no abnormalities. At 2 years of age he was diagnosed with childhood autism (Autism Diagnostic Observation Schedule type G (ADOS-G); Communication score: 5, Social score: 14). At 8 years of age a WISC-III test showed a very uneven profile with specific non-verbal visio-spatial difficulties (verbal IQ = 103, performance IQ = 60, global IQ = 79). Currently, he attends a class for children with special needs in a normal primary school. Verbally he is highly skilled in both Danish and English.

Periodic idiopathic trembling was noted from the age of 2 days and according to the mother it persisted for the first 5 weeks. This description is in accordance with a diagnosis of benign neonatal convulsions but this was never diagnosed. Currently, he has no epilepsy diagnosis; however, according to the parents he has brief episodes of non-responsiveness resembling absence seizures. Consequently, electroencephalographic (EEG) examination was carried out at ages 5 and 9 years during sleep, hyperventilation, photo-stimulation, and during periods of non-responsiveness but no abnormalities were observed. Cerebral magnetic resonance (MR) scanning of the brain at age 8 years was normal.

The National Ethics Committees and the Danish Data Protection Agency approved the study, and informed consent was obtained from the parents.

### Patients and control individuals for mutation screening of KCNQ3

Mutation screening of *KCNQ3* was performed in two steps. As a first step DNA from a cohort comprising 100 Portuguese and 48 Danish ASD patients were screened for *KCNQ3* mutations by direct sequencing. The Portuguese ASD patients were recruited at the Hospital Pediátrico de Coimbra and all originated from mainland Portugal and the Azorean islands. The male-female ratio was 4.8:1, and the ages ranged between 2 and 18 years (mean age 6.8 years). Idiopathic subjects were included after clinical assessment and screening for known medical and genetic conditions associated with autism, including testing for Fragile X mutations (FRAXA and FRAXE), chromosomal abnormalities, neurocutaneous syndromes, endocrine (thyroid function screening), and metabolic disorders. About 35 of the 48 Danish ASD patients were recruited at child psychiatric hospitals in the western part of Denmark (Jutland) (age range 3–30 years, with mean age of 10 years and male-female ratio of 3:1). Seven autistic patients were ascertained at the Kennedy Center (Glostrup, Denmark) (age range 13–37 years, mean age 20.4 and male-female ratio of 2.5:1). These patients were unrelated and part of the IMGSAC group and accordingly some of the patients had siblings and some even additional relatives with a diagnosis of pervasive developmental disorder. These patients were screened for chromosomal abnormalities and fragile X syndrome and a physical examination included a careful search for phakamatoses to rule out Tuberous Sclerosis (TSC). Four patients diagnosed within the autism spectrum were collected at the Psychiatric Hospital in Hillerød (Frederiksborg Amt, Denmark). In addition, two DNA samples (one male, one female) from individuals diagnosed within the ASD spectrum and with chromosomal rearrangements were included in the screening. These samples were collected at the Wilhelm Johannsen Centre for Functional Genome Research, University of Copenhagen (Denmark). In all of the above ASD patients diagnosis was made in accordance with DSM-IV or ICD-10 criteria using ADI-R in addition to ADOS or the Childhood Autism Rating Scale.

The c.1720C > T variant in *KCNQ3* was first identified in one Portuguese ASD patient (patient B, Table 1) by direct sequencing. As a next step 271 additional Portuguese ASD patients fulfilling the same criteria as the first cohort were specifically screened for the c.1720C > T variant by a PCR/enzyme cleavage assay whereby two additional patients (patient C and D) were identified as carriers of the c.1720C > T variant. Hence, a total of 419 ASD patients (371 Portuguese- and 48 Danish ASD patients) were screened for the c.1720C > T variant. The three male Portuguese patients (Patient B, C, D in Table 1) were diagnosed with childhood autism using the Autism Diagnostic Interview-Revised (ADI-R) and ADOS (Lord et al., 1994) and had no history of convulsions. The inheritance pattern of the c.1720C > T variant was ascertained both by direct sequencing of exon 13 of *KCNQ3* and by the PCR/enzyme cleavage assay in patient B, C, and D and their parents. As controls 96 Caucasian individuals from the Human Random Control DNA panel (HRC-1, Sigma-Aldrich, St. Louis, USA), and 100 Portuguese individuals without neuropsychiatric disease (self reported) from blood donor centers throughout Portugal were included.

**Table 1:** **Clinical description of three Portuguese individuals carrying a c.1720C > T variant and diagnosed with childhood autism**.

	Patient B	Patient C	Patient D
Current age	13 years	14 years	7 years
Born at gestational week	36	41	40
Apgar scores	10/5	8/5	9/5
Birth length, weight, head circumference	49 cm, 3760 g, 36 cm	50 cm, 3955 g, 37 cm	51 cm, 4290 g, 37,3 cm
Dysmorphic features	No	No	No
Walking age	13 months	16 months	23 months
Age at first word	16 months	24 months	18 months
Age at first sentence	48 months	Still not capable	Still not capable
Current height, weight, head circumference	50th percentile, 75th percentile, +2SD	25th percentile, 25th percentile, −2SD	50th percentile, 90th percentile, + 2SD
Neuronal examination	Normal	Normal	Normal
Mental capacity	WISC-III: verbal IQ 97, performance IQ 84, global IQ 88	GDE (Griffiths, 1984): verbal IQ 46, performance IQ 63, global IQ 61	GDE: verbal IQ 31, performance IQ 67, global IQ 58
SNP inherited from	Father	Mother	Father
Family history	No neurological- or psychiatric disorders	Mother: Major depression	No neurological- or psychiatric disorders

### Cytogenetic analyses, fluorescence *in situ* hybridization and array-comparative genomic hybridization

Cytogenetic analysis and fluorescence *in situ* hybridization (FISH) was performed according to standard protocols, and array-based comparative genome hybridization was performed as previously described (Erdogan et al., 2006).

### Whole genome amplification

When necessary, genomic DNA was uniformly amplified using GenomiPhi™ DNA Amplification Kit (GE Healthcare, Buckinghamshire, UK).

### Mutation screening of KCNQ3

All 15 coding exons and intron-exon boundaries of *KCNQ3* (NM_004519.3) were amplified by PCR. Sequencing reactions were carried out using BigDye^®^ Terminator v 1.1 Cycle Sequencing Kit (Life Technologies, California, USA) and analyzed by an ABI 3100 AVANT Genetic Analyzer (Life Technologies, California, USA). ChromasPro version 1.33 (Technelysium Pty Ltd, Australia) was used to visualize the data. Nucleotide changes were verified by a second PCR amplification of non-genome amplified patient DNA, sequencing and restriction cleavage.

### Restriction enzyme assay for detection of c.1720C > T (p.P574S) in KCNQ3

A PCR product of 461 base pairs (bp) encompassing exon 13 of *KCNQ3* was amplified using primers KCNQ3_13a: TATTCCAAACCCTTATCTCAT and KCNQ3_13b: AAACAGGTGGGG CTATTA. PCR fragments amplified from the WT allele were digested into two fragments with lengths 438 and 23 bp by the restriction enzyme *Hpy*188III, whereas PCR fragments amplified from the c.1720C > T allele were digested into three fragments with lengths 337, 101, and 23 bp.

### Expression plasmids and cloning

The plasmids hK_V_7.2-hK_V_7.5 in pXOOM or pXOON, hK_V_7.3-flag in pNS2z, and hK_V_7.2-cmyc in pNS2z used in this study have been described previously (Bentzen et al., 2006; Rasmussen et al., 2007). K_V_7.4 cDNA was amplified with PCR and inserted into pNS2z to generate C-terminally myc-tagged K_V_7.4. To generate the extracellularly tagged expression plasmid hK_V_7.5-3xHA in pXOOM, 3 HA-tags were inserted into the TM3-TM4 linker of hK_V_7.5 by PCR using the primers 5′-CCAGATTACGCGTACCC TTACGACGTTCCAGATTACGCTGGTAATATTTTTGCCAC-3′ and 5′-GACATCGTAT GGGTAAGCGTAGTCTGGGACGTCGTATGGGTACTGAGTTTTTGCAGAAAC-3′. Human CD4-WT in pcDNA3.1 was a kind gift from James Trimmer (University of California Davis, CA, USA) and has been described earlier (Gu et al., 2003). The chimera hCD4-hK_V_7.3CT in pcDNA3.1 was generated using standard PCR and in-frame insertion of cDNA corresponding to K_V_7.3 amino acids 358–873 into *Not*I and *Xho*I sites of the wild-type (WT) construct. The point mutation c.1720C > T leading to the amino acid exchange P574S was introduced using mutated oligonucleotide extension (*Pfu*Turbo Polymerase, Stratagene, La Jolla, CA, USA) from the plasmid template harboring the cDNA of interest, digested with *Dpn*I (Fermentas, St. Leon, Germany) and transformed into *E. coli* XL1 Blue cells. All plasmids were verified by complete DNA sequencing of the cDNA insert (Macrogen Inc., Seoul, Rep. of Korea). The Gene Bank Accession numbers of the human cDNAs are: NM_004519 (K_V_7.3), NM_004518 (K_V_7.3, isoform c), NM_004700 (K_V_7.4), and NM_019842 (K_V_7.5). Protein accession number for K_V_7.3 is NP_004510.

### Heterologous expression in *Xenopus laevis* oocytes

#### *In vitro* transcription

The cRNA were prepared from linearized hK_V_7.2, hK_V_7.3 WT and mutant, K_V_7.4, and K_V_7.5 constructs in pXOOM or pXOON using the Ambion T7 m-Message Machine kit according to the manufacturer’s instructions (Ambion, Austin, TX, USA). RNA concentrations were determined by UV spectroscopy, integrity was confirmed by gel electrophoresis. cRNAs were stored at −80°C until injection.

#### Oocyte isolation and injection

Female *Xenopus laevis* frogs were anesthetized with Tricain (2 g/l, Sigma, Brøndby, Denmark) and ovarian lobes were removed. Oocytes were defolliculated enzymatically in 1% collagenase (Boehringer Mannheim/Roche, Hvidovre, Denmark) and 0.1% trypsin inhibitor (Sigma) in Kulori’s solution for 1 h followed by wash in Kulori’s solution (in mM: 90 NaCl, 4 KCl, 1 MgCl_2_, 1 CaCl_2_, 5 Hepes, pH 7.4) containing 0.1% BSA (Sigma). Oocytes were injected using a Nanoject microinjector (Drummond Scientific, Broomall, PA, USA) with 1 ng hK_V_7.2, 7.4, or 7.5 mixed with hK_V_7.3 WT or hK_V_7.3_P574S cRNA (in a 1:1 molar ratio) diluted in 50 nl diethylpyrocarbonate treated water. Oocytes were kept in Kulori’s solution at 19°C.

#### Two-electrode voltage-clamp recordings

Currents were recorded at room temperature by two-electrode voltage-clamp (TEVC) 2 days after injection using a Dagan 2B amplifier (Clampator 1, Dagan, Chicago, IL, USA). The oocytes were perfused with Kulori’s solution and pipettes were pulled from borosilicate glass and had a final tip resistance of 0.5–2.5 MΩ when filled with 2 M KCl. Data were acquired using Pulse software (HEKA electronics, Germany) and analyzed with Igor (WaveMetrics, Lake Oswego, OR, USA) and GraphPad Prism (GraphPad Software, San Diego, CA, USA). All experiments were performed in 3–4 different batches of oocytes.

#### Data analysis

Data are presented as mean ± standard error of the mean. For statistical analyses ANOVA combined with Student-Newman-Keuls post test was used and *p* < 0.05 was considered significant (*).

### Cell cultures and transfections

HEK 293 cells were grown in DMEM (Invitrogen, Glostrup, Denmark) supplemented with 100 U/ml penicillin, 100 mg/ml streptomycin and 10% FCS (Sigma-Aldrich, Copenhagen, Denmark) at 37°C in a humidified atmosphere with 5% CO_2_. Transfections were carried out using the Lipofectamine-Plus Reagent system (Invitrogen) according to the manufacturer’s instructions. Hippocampal cultures were prepared as previously described (Rasmussen et al., 2007) and transfected at 7–8 days *in vitro* (DIV) using the lipofectamine 2000 method (Invitrogen) with a total of 0.9 μg of DNA per cover-slip. Transfection was carried out for 1 h at 37°C, 5% CO_2_ after which the neurons were transferred back to the dishes containing the glial cell layer. Neurons were left for expression for 48 h.

### Immunocytochemistry

HEK 293 cells or primary hippocampal neurons were fixed in 3% paraformaldehyde in PBS for 15–20 min at room temperature. Blocking and permeabilization was performed by a 30 min incubation with 0.2% fish skin gelatin in phosphate buffered saline supplemented with 0.1% Triton X-100 (PBST). The cells were then incubated for 1 h in primary antibodies diluted in PBST. Primary antibodies employed were: rabbit anti-c-myc (A-14, 1:50 dilution, Santa Cruz Biotechnology, Heidelberg, Germany), mouse anti-FLAG (M2, 1:250 dilution, Sigma-Aldrich, Copenhagen, Denmark), and rat anti-HA (3F10, 1:50 dilution, Santa Cruz Biotechnology). For immunofluorescent detection, Alexa Fluor^®^–coupled secondary antibodies were diluted in PBST and applied for 45 min. The cover-slips were mounted in Prolong Gold (Invitrogen, Glostrup, Denmark).

### Confocal microscopy and imaging

Laser scanning confocal microscopy was performed using the Leica TCS SP2 system equipped with argon and helium-neon lasers. Images were acquired using a 63× water immersion objective, NA 1.2 with a pinhole size of 0.8-1 and a pixel format of 1024 × 1024. Line averaging was used to reduce noise. For double- and triple-labeling experiments sequential scanning was employed to allow the separation of signals from the individual channels. Acquired images were treated using Adobe Photoshop CS4 and Adobe Illustrator CS4.

## Results

### Identification of a de novo reciprocal translocation *t*(3;8) (q21;q24) disrupting *KCNQ3*

**Figure 1:**
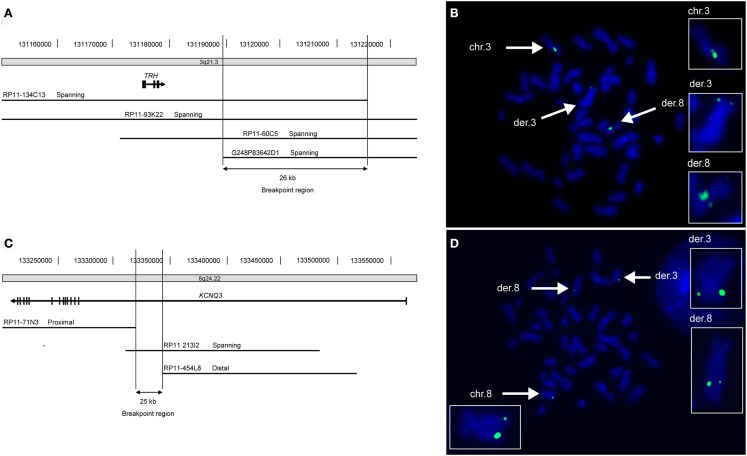
**FISH mapping of translocation breakpoints reveals truncated KCNQ3 gene in patient A**. **(A)** Schematic depiction of the 26 kb breakpoint region within chromosomal region 3q21.3 of patient A. The breakpoint region is located 10.5 kb downstream of the *TRH* gene and contains no genes. **(B)** Picture showing metaphase chromosomes from patient A (blue) hybridized with the chromosome 3 specific probe RP11-93K22 (green) that spans the breakpoint. The normal chromosome 3 as well as both derivative chromosomes are marked with white arrows and enlarged in the boxes to the right. **(C)** The 25 kb breakpoint region on chromosome 8 lies within intron 1 of the *KCNQ3* gene. **(D)** The chromosome 8 specific probe RP11-213I2 (green) spans the breakpoint. The normal chromosome 8 as well as the two derivative chromosomes are marked with white arrows and enlarged.

By cytogenetic analysis a *de novo* *t*(3;8) (q21;q24) translocation was identified in Patient A. The breakpoints of this translocation were mapped using FISH. The breakpoint on chromosome 3 was delineated to a 26 kb region (chr3:131.189.991-131.216.096; NCBI36/hg18) at 3q21.3 located 10.5 kb downstream of the thyrotropin-releasing hormone (THR) gene (Figure 1). No annotated human genes or mRNAs are located within this breakpoint region. On chromosome 8q24.22 the breakpoint was localized in a 25 kb region (chr8:133.318.034-133.343.450; NCBI36/hg18) within exon 1 of the *KCNQ3* gene (Figure 1). No disease-related copy number variations were identified by array comparative genomic hybridization (CGH) in this patient.

### Identification of a rare variant c.1720C > T in *KCNQ3* in three unrelated ASD patients

**Figure 2:**
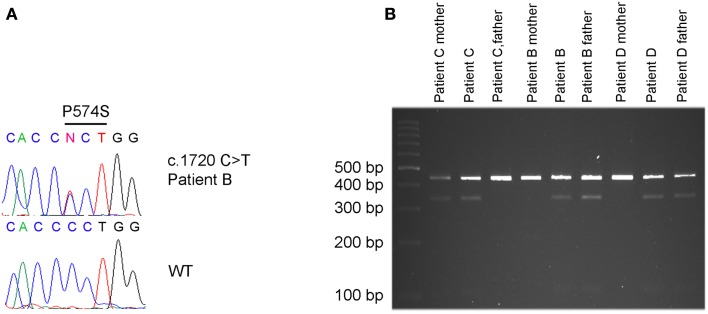
**c.1720C > T [p.P574S] variant detected in patient B, C and D**. **(A)** The c.1720 C > T mutation in patient B was identified by Sanger sequencing. **(B)** The same mutation was identified in patient C and his mother, in patient D and his father and confirmed in patient B and his father by restriction enzyme cleavage of a PCR product encompassing the mutation.

A paternally inherited c.1720C > T missense mutation in exon 13 of *KCNQ3* was identified in patient B by direct sequencing (Figure 2A). There is no history of psychiatric- or neurological disorders in this family. The mutation results in an amino acid change at position 574 replacing proline by serine (p.P574S). By restriction enzyme assay the c.1720C > T (p.P574S) variant in *KCNQ3* was identified in two additional ASD patients (patient C and D) (Figure 2B) and was confirmed in patient B. Patient C inherited the variant from the mother who suffers from major depression and patient D inherited the variant from the father who does not suffer from any psychiatric- or neurological disorders (Figure 2B). The c.1720C > T mutation in patients C and D was confirmed by direct sequencing of a second PCR product from non-amplified DNA. No c.1720C > T mutations were identified in 96 UK Caucasian and 100 Portuguese controls.

### The P574S substitution in K_V_7.3 reduces current through the K_V_7.3/K_V_7.5 complex

**Figure 3:**
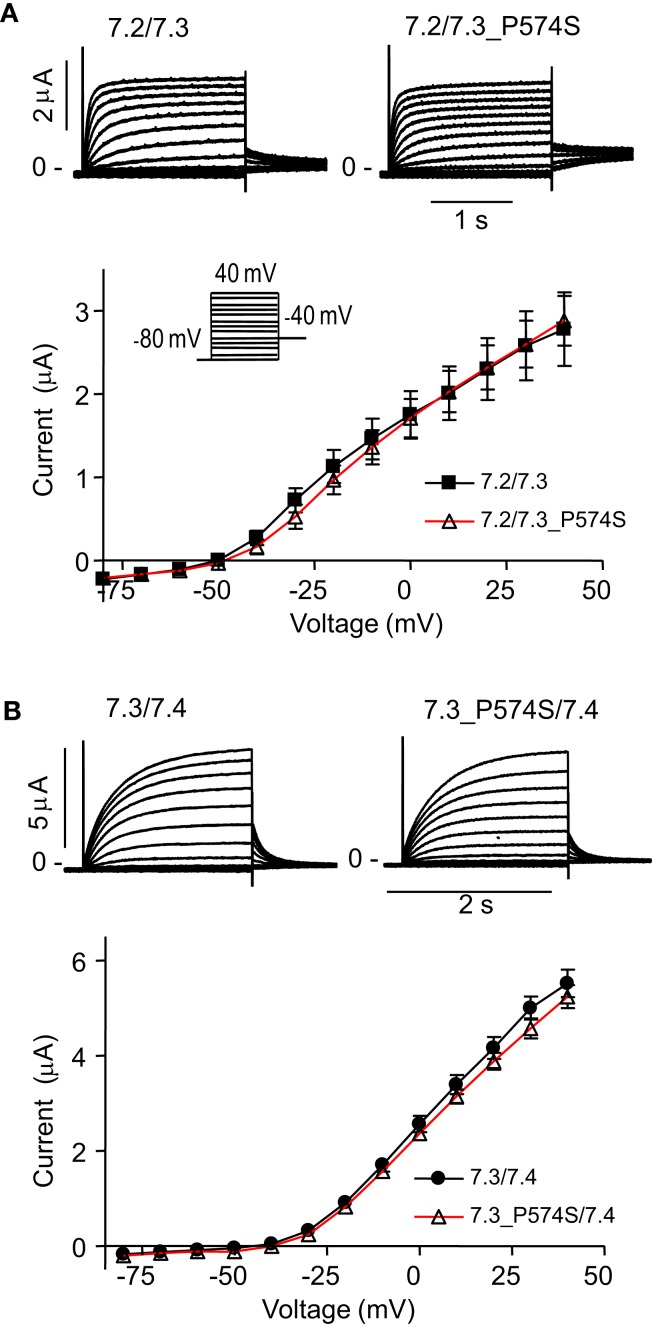
**Effect of K_V_7.3_P574S on K_V_7.2 and K_V_7.4 in *X. laevis* oocytes**. Currents were activated by voltage-steps from −80 mV to +40 mV in 10 mV increments. Representative currents are shown as well as steady-state current plotted as a function of voltage. **(A)** K_V_7.2 was co-expressed with either K_V_7.3 WT (*n* = 6) or P574S (*n* = 5). **(B)** Effect of K_V_7.3___P574S on K_V_7.4. K_V_7.4 was co-expressed with either K_V_7.3 WT (*n* = 10) or P574S (*n* = 13) in *X. laevis* oocytes.

**Figure 4:**
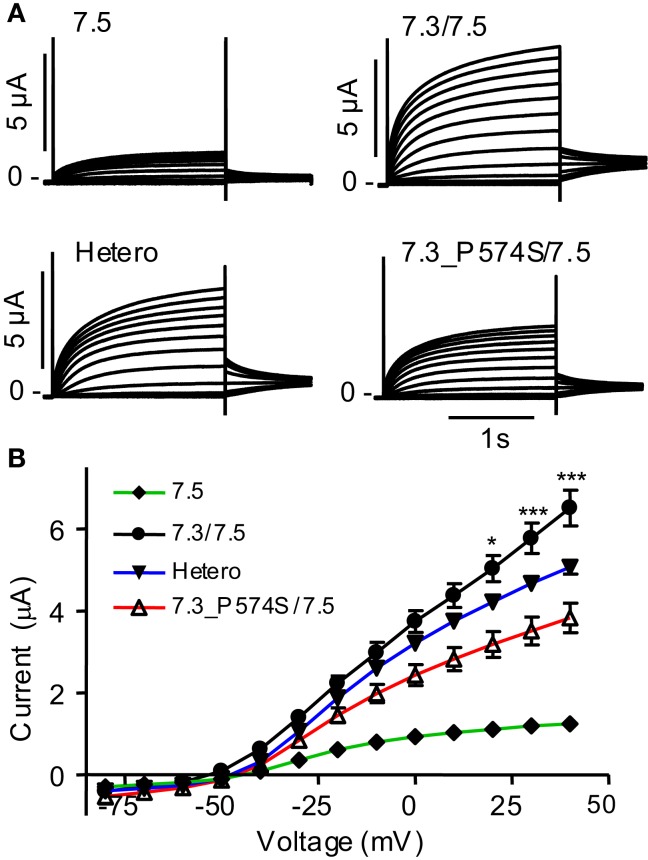
**Effect of K_V_7.3_P574S on K_V_7.5**. K_V_7.5 was expressed alone (*n* = 8) or co-expressed with either K_V_7.3 WT (*n* = 7), P547S (*n* = 6), or K_V_7.5 mixed with K_V_7.3 WT or K_V_7.3___P574S (in a 1:1 molar ratio, Hetero, *n* = 6) in *X. laevis* oocytes. Currents were activated by voltage-steps from −80 to +40 mV in 10 mV increments. **(A)** Representative currents are shown. **(B)** Steady-state current plotted as a function of voltage. Asterisks indicate statistical difference between Hetero and K_V_7.3/K_V_7.5. Comparison of the other points were left out for clarity and for voltages higher than 0 mV, all points were statistically different.

To address effects of the mutation P574S on ion channel function, we heterologously expressed mutant channels in *Xenopus laevis* oocytes. Since K_V_7.3 does not form functional channels on its own (Schwake et al., 2000), we investigated whether the K_V_7.3___P574S mutation could affect the function of other neuronal members of the K_V_7 family. K_V_7.3_P574S or K_V_7.3 WT was co-expressed with K_V_7.2, K_V_7.4, or K_V_7.5 in *Xenopus laevis* oocytes and currents were recorded by TEVC. In agreement with Neubauer et al. (2008), we found that current levels for K_V_7.2/K_V_7.3_P574S were similar to those of K_V_7.2/K_V_7.3 (Figure 3A). Similarly, the function of K_V_7.4 channels did not appear to be affected by the mutation, as oocytes expressing K_V_7.3___P574S/K_V_7.4 had similar current levels as K_V_7.3/K_V_7.4 (Figure 3B). In a final set of experiments, we tested the effect of K_V_7.3___P574S on K_V_7.5 currents. In line with previous work by Lerche et al. (2000), co-expression of K_V_7.5 with K_V_7.3 dramatically increased current levels compared to K_V_7.5 alone (Figure 4). Expression of K_V_7.3___P574S also enhanced K_V_7.5 current levels but to a significantly lesser extent than WT K_V_7.3. These results suggest that K_V_7.3___P574S has not lost its ability to interact with K_V_7.5. Since both patients B and C were heterozygous for the K_V_7.3___P574S mutation, we mimicked the heterozygous state by co-expressing K_V_7.5 with K_V_7.3 and K_V_7.3___P574S in a 2:1:1 ratio. The resulting current levels were intermediate of that of K_V_7.3/K_V_7.5 and K_V_7.3___P574S/K_V_7.5, indicating that (1) K_V_7.3___P574S is not dominant-negative, and (2) co-expression of WT does not rescue the K_V_7.3___P574S phenotype. The difference in current levels between the heterozygote and WT K_V_7.3/K_V_7.5 is statistically significant as indicated by the asterisk in Figure 4B. Meticulous inspection of the curves in Figure 4B reveals that for K_V_7.5 expressed alone, there is a tendency for the current-voltage relationship to flatten out at higher voltages and this tendency appears to be removed by co-expression with K_V_7.3, making the current-voltage curve more linear.

### The P574S substitution in K_V_7.3 does not affect trafficking in HEK 293 cells and neurons

**Figure 5:**
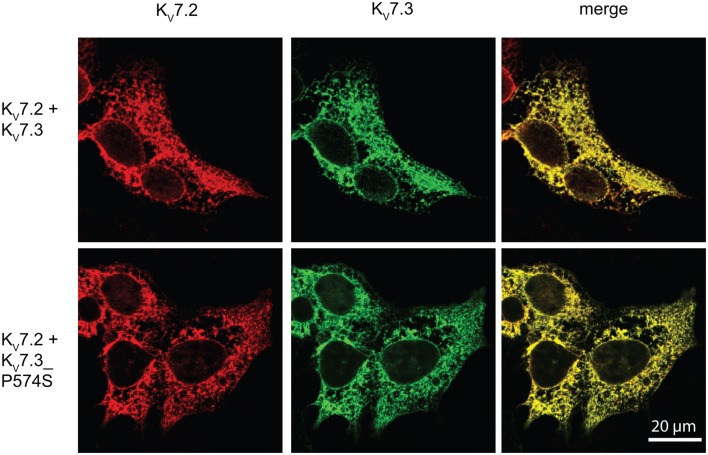
**No effect of K_V_7.3___P574S on localization of the K_V_7.2/K_V_7.3 complex in HEK 293 cells**. K_V_7.2 was transiently expressed in HEK 293 cells together with K_V_7.3 WT or K_V_7.3___P574S, and the localization of the complex was analyzed by immunocytochemistry and confocal microscopy. As illustrated, the P574S mutation is without effect on the localization of the complex that displays a primarily intracellular localization pattern. Scale bar 20 μm.

Since the P574S mutation reportedly is without effect upon the current characteristics of the K_V_7.2/K_V_7.3 complex (Miceli et al., 2009), we decided to investigate whether the P574S mutation could affect the localization of the heteromeric K_V_7.2/K_V_7.3 complex. We first analyzed the localization of K_V_7.2 and K_V_7.3 upon co-expression in HEK 293 cells. As illustrated in Figure 5, both channel subunits displayed a primarily intracellular staining pattern. The subunits appeared to co-localize to a large degree in the intracellular structures and only weak staining could be detected in association with the cell surface. Importantly, co-expression of K_V_7.2 and K_V_7.3___P574S resulted in a staining pattern that was indistinguishable from the co-expression of the WT channels. Thus, K_V_7.3___P574S does not appear to have an impact on the localization of the K_V_7.2/K_V_7.3 heteromeric complex in HEK 293 cells.

In neurons, the K_V_7.2/K_V_7.3 complex is localized to the AIS (Devaux et al., 2004; Chung et al., 2006; Rasmussen et al., 2007). We therefore speculated that the specific localization of the complex to the AIS could be disturbed by the P574S mutation. To address this question, we first examined the localization of K_V_7.3 and K_V_7.3___P574S upon exogenous expression in cultured rat hippocampal neurons. As previously reported, singly expressed K_V_7.3 was primarily observed intracellularly with no significant enrichment in the AIS (Rasmussen et al., 2007). Likewise, K_V_7.3___P574S demonstrated a primarily intracellular staining pattern similar to the WT subunit.

**Figure 6:**
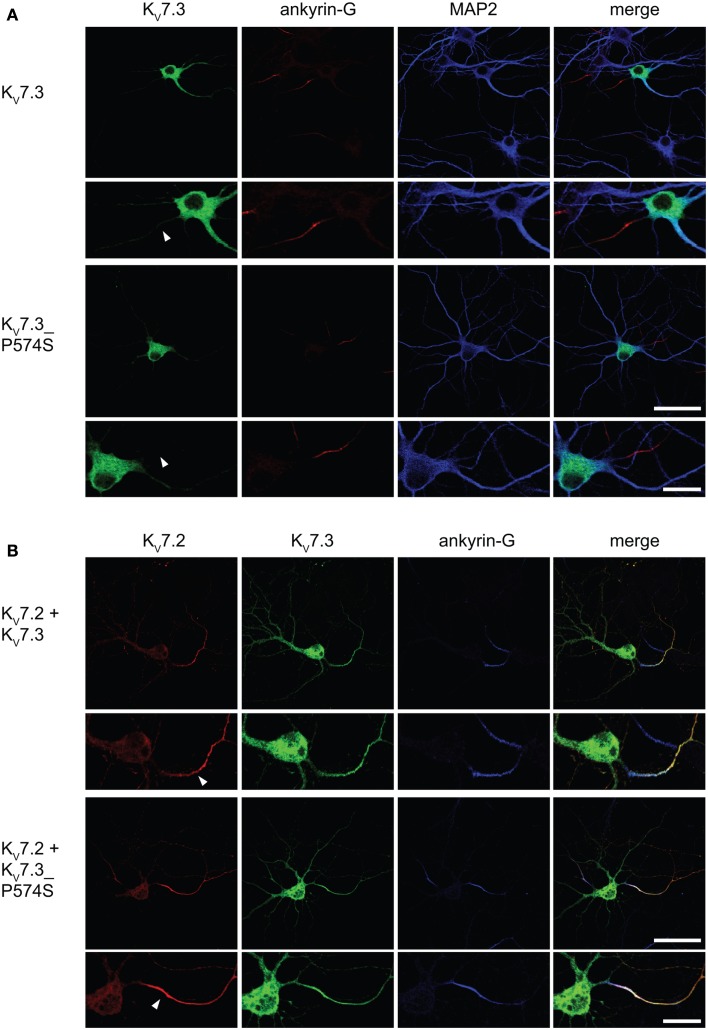
**K_V_7.2/K_V_7.3___P574 complexes still target to the AIS of cultured hippocampal neurons**. Confocal images of cultured rat hippocampal neurons (10 DIV) expressing K_V_7.3 WT or K_V_7.3___P574S **(A)** and co-transfected with K_V_7.2 **(B)**. **(A)** K_V_7.3 is primarily intracellularly expressed when expressed on its own. No significant K_V_7.3 expression is observed at the AIS. **(B)** When co-expressing K_V_7.3 with K_V_7.2, both channel subunits are observed at the AIS. K_V_7.3___P574S displays the same localization characteristics. White arrowhead points to the location of the AIS. Ankyrin-G: marker of the axon initial segment, MAP2: marker of the somatodendritic region of neurons. Scale bars 50 and 20 μm, respectively.

**Figure 7:**
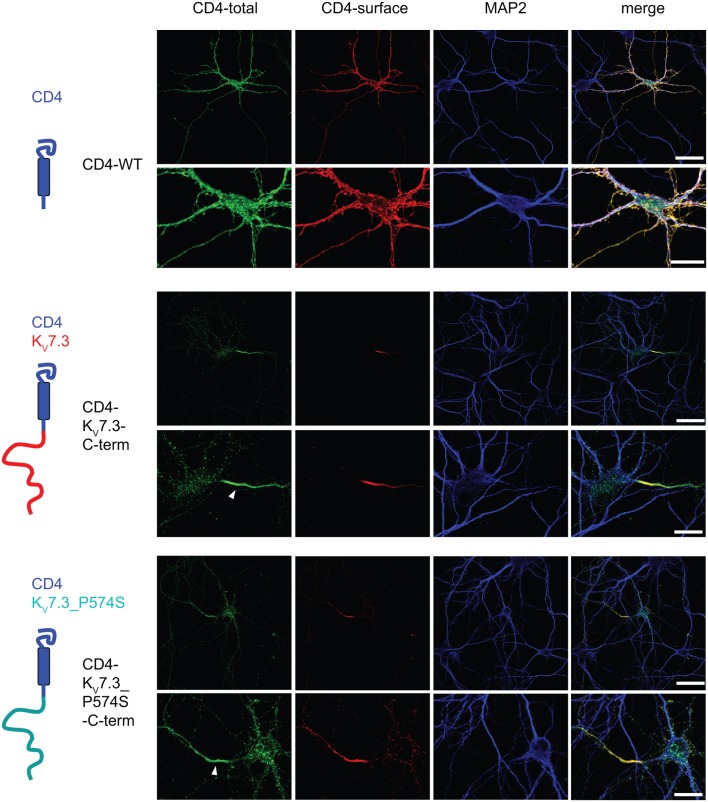
**CD4-K_V_7.3Cterm___P574 can target to the AIS**. Confocal images of cultured rat hippocampal neurons (10 DIV) transfected with CD4 (two upper panels), CD4-K_V_7.3 C-term (two middle panels) and CD4-K_V_7.3___P574S C-term (two lower panels). The panels to the left illustrate the structure of the chimeric constructs analyzed. CD4-total reflects total CD4 staining in permeabilized cells. CD4-surface is a surface staining of the same cells where the CD4 antibody was applied before permeabilization. The somatodendritic marker MAP2 was included to indicate the location of the AIS (neurite which is MAP2 negative). As expected, CD4 distributes in a non-polarized manner on the surface of axon, soma and dendrites. When the C-terminal of K_V_7.3 is attached to CD4, the reporter redistributes to the axon initial segment, which illustrates that the K_V_7.3 C-terminal contains information for AIS localization. The C-terminus of K_V_7.3___P574S still has the ability to direct CD4 to the AIS. White arrowhead points to the location of the AIS. Scale bars 50 and 20 μm, respectively.

Upon co-expression of K_V_7.2 and K_V_7.3, the channel complex appears in the AIS (Rasmussen et al., 2007). To investigate whether the K_V_7.3___P574S mutation perturbed the localization of the complex to the AIS, we transiently expressed K_V_7.2 with either WT K_V_7.3 or K_V_7.3___P574S in cultured hippocampal neurons. As illustrated in Figure 6B, the P574S mutation did not impair the localization of the K_V_7.2/K_V_7.3___P574S complex as it localized to the AIS similar to the WT complex. These results were further emphasized by experiments using chimeric constructs of the transmembrane protein CD4 and K_V_7.3/K_V_7.3___P574S. We have previously demonstrated that the ability of K_V_7.3 to direct the K_V_7.2/K_V_7.3 complex to the AIS critically depends on an ankyrin-G binding sequence in the C-terminal tail of K_V_7.3 (Rasmussen et al., 2007). We therefore attached the C-terminal tail of K_V_7.3 to a truncated version of the CD4 receptor to examine whether this part of K_V_7.3 would be sufficient to redirect the otherwise non-polarized protein CD4 to the AIS (Figure 7, left panel). As expected, the truncated version of CD4 displayed a non-polarized localization pattern upon expression in cultured hippocampal neurons (Figure 7, top panel). Attachment of the K_V_7.3 C-terminus was sufficient to drive an AIS localization of the chimera (Figure 7, middle panel). As illustrated, introduction of the P574S mutation into the chimera was without effect as the mutated chimera was still able to target efficiently to the AIS (Figure 7, lower panels).

**Figure 8:**
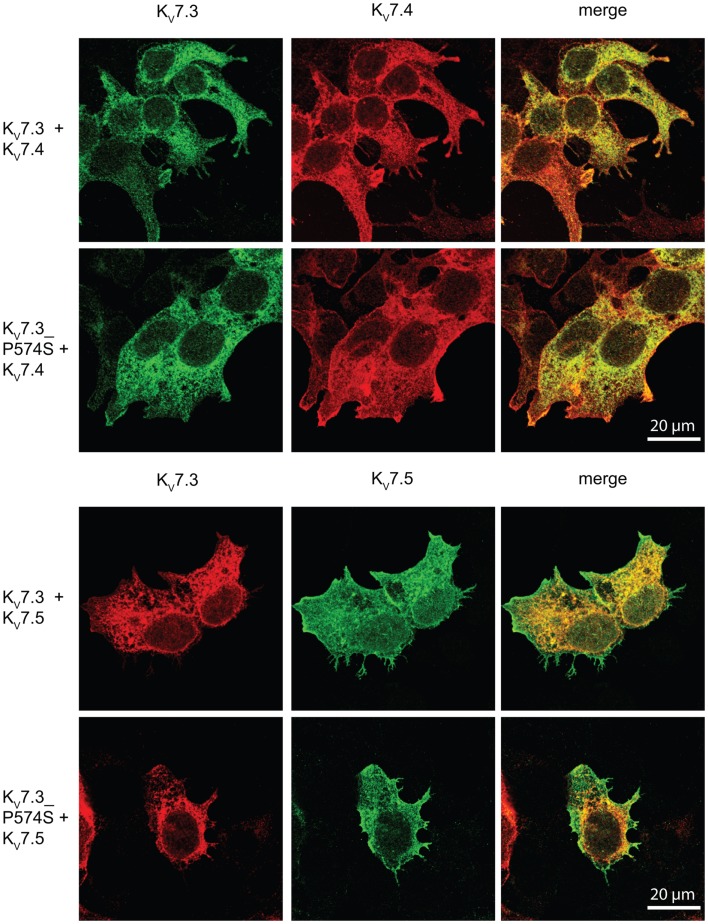
**No effect of K_V_7.3___P574S on the localization of K_V_7.4 and K_V_7.5 containing complexes in HEK 293 cells**. K_V_7.4 and K_V_7.5 were transiently expressed in HEK 293 cells together with K_V_7.3 or K_V_7.3___P574S and the localization of the expressed subunits analyzed by immunocytochemistry and confocal microscopy. As illustrated, the P574S mutation is without effect on the localization pattern of the complexes that displays a mixed surface and intracellular staining pattern. Scale bar 20 μm.

Since the P574S mutation did not affect the localization of the classical K_V_7.2/K_V_7.3 complex, we investigated the impact of the mutation on the localization of heteromeric channels including the K_V_7.4 or K_V_7.5 subunits. We transiently co-expressed WT K_V_7.3 or the mutant P574S with either the K_V_7.4 or the K_V_7.5 subunit in HEK 293 cells and analyzed the localization of the subunits by confocal microscopy. As illustrated in Figure 8, K_V_7.3/K_V_7.4 and K_V_7.3/K_V_7.5 complexes demonstrated a mixed surface and intracellular staining pattern demonstrating that both K_V_7.4 and K_V_7.5 subunits can pull a fraction of K_V_7.3 subunits to the cell surface. However, the same localization pattern was observed when analyzing the mutant complexes. Thus, the P574S mutation was without significant effect on the localization of K_V_7.4 and K_V_7.5 containing complexes.

## Discussion

Mutations in *KCNQ3* (and *KCNQ2*) have been previously described in patients with rolandic epilepsy and IGE (Neubauer et al., 2008) including benign neonatal convulsions. A considerable proportion of patients with these types of epilepsies also have ID and/or behavioral problems (ADHD, ASD, anxiety, depression) (Borgatti et al., 2004; Steinlein et al., 2007; Akanuma et al., 2008; Tovia et al., 2011) which supports a common genetic etiology and accordingly suggest *KCNQ3* (and *KCNQ2)* as candidate susceptibility genes for ID and various psychiatric disorders. This is substantiated by a *KCNQ2* knock-out mouse model that shows spontaneous seizures and behavioral hyperactivity (Peters et al., 2005); by finding of two patients with psychomotor retardation and convulsions with a 8,35 Mb deletion encompassing *KCNQ3* (Verheij et al., 2009); and by the association of markers close to *KCNQ3* with bipolar disorder (Avramopoulos et al., 2004; Zandi et al., 2008; Zhang et al., 2010).

In line with this hypothesis we here demonstrate different *KCNQ3* alterations (truncating mutation, rare SNP with abnormal electrophysiological profile) in four patients with childhood autism and in one transmitting parent with major depression.

The c.1720C > T [p.P574S] nucleotide change was identified in three unrelated Portuguese patients with childhood autism. In two cases (patients B and D) the variant was inherited from an apparently normal parent and in the third case (patient C) transmitted from a mother with major depression. This nucleotide change is now annotated as a rare SNP in dbSNP (rs74582884, Minor Allele Frequency *A* = 0,012) and was previously reported in 2 of 62 patients with rolandic epilepsy and in 8 of 455 patients with IGE but not in 454 healthy controls (Neubauer et al., 2008). Both patients with rolandic epilepsy inherited the mutation from a healthy parent. This raises the possibility that the rs74582884 SNP conveys liability for general psychopathology but at the same time suggests that additional genetic and/or environmental factors may have an impact on the phenotypical outcome of carriers. Indeed, the same SNP was reported in a patient with benign familial neonatal seizures who, in addition, carried a *de novo* mutation in *KCNQ2* that changed channel gating. Since the SNP in *KCNQ3* was inherited from a father and a paternal grandmother without neurological abnormalities the authors suggested that the SNP was not responsible for the observed epilepsy (Miceli et al., 2009). However, our data suggest the c.1720C > T nucleotide change as a contributing factor. Combining our data with all published sequencing studies of *KCNQ3* shows that rs74582884 is a rare variant as it is absent in a total of 700 controls (Neubauer et al., 2008; Miceli et al., 2009).

**Figure 9:**
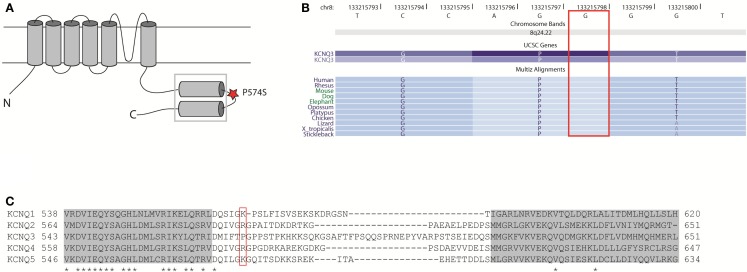
**Localization and conservation of the SNP c.1720C > T [p.P574S] in *KCNQ3***. **(A)** The K_V_7 subunits consist of six transmembrane domains and a long intracellular carboxy-terminal that contains the subunit interaction domain (*sid*) (within the square). The P574 amino acid is located in the linker region between two coiled-coil regions. Adapted from (Wehling et al., 2007). **(B)** The c.1720C nucleotide is conserved between species. Adapted from the UCSC genome browser hg18. **(C)** Amino acid alignment of the *si* domains from all K_V_7 channels. The two coiled-coil domains are depicted as gray boxes and conserved amino acids are marked with an asterisk. The P574 amino acid is located in the linker region between two coiled-coil regions and is not conserved between K_V_7 members. Adapted from Wehling et al. (2007).

The proline P574 is located in the linker region between two subunit interaction domains in the C-terminal region of K_V_7.3 (Figure 9A). This part of the protein is involved in subunit assembly, maturation, and transport of channels (Schwake et al., 2006). The amino acid residue P574 is evolutionally conserved (Figure 9B), but it is not conserved among the K_V_7 family members of voltage-gated potassium channels (Figure 9C). This indicates that P574 is important for K_V_7.3 function and this function is probably not shared by the other K_V_7 members. Since K_V_7.3 is the most promiscuous of the K_V_7 proteins forming heterotetrameric channels with K_V_7.2 (Schroeder et al., 1998), K_V_7.4 (Kubisch et al., 1999), and K_V_7.5 (Schroeder et al., 2000) we investigated the effect of the P574S change on the localization of and current through heteromeric K_V_7.2/K_V_7.3_P574S, K_V_7.4/K_V_7.3_P574S, and K_V_7.5/K_V_7.3_P574S channels.

There was no significant effect of the P574S amino acid change on the localization of K_V_7.3 containing channel complexes neither in HEK 293 cells (Figures 5 and 8) nor in cultured rat hippocampal neurons (Figure 6). The data further show that subunit assembly as well as AIS localization were unaffected by the mutation (Figure 7).

Currents elicited in *X. laevis* oocytes upon expression of K_V_7.2/K_V_7.3_P574S did not differ from WT currents (Figure 3A) in agreement with previous reports (Neubauer et al., 2008; Miceli et al., 2009). Likewise, we did not observe any changes in the current mediated by K_V_7.4/K_V_7.3_P574S complexes (Figure 3B). However, co-expressing K_V_7.3_P574S with K_V_7.5 reduced the current significantly compared to WT K_V_7.3 (Figure 4), possibly due to altered inactivation properties. Mimicking the heterozygous state of the patients showed intermediate current amplitudes indicating that K_V_7.3_P574S does not have a dominant-negative effect; however, the effect of the mutation is not rescued by co-expression of WT K_V_7.3. These results show, for the first time, how the rs74582884 SNP in *KCNQ3* identified in patients with ASD, ID, major depression or various types of epilepsy functionally impairs the function of a channel complex formed by K_V_7.3/K_V_7.5 complexes. Accordingly, *KCNQ3* and *KCNQ5* (OMIM 607357) are suggestive susceptibility genes for ASD, ID, major depression, epilepsy, and due to the considerable overlap in etiologies also for other psychiatric disorders like ADHD, bipolar disorder, and anxiety disorder. To our knowledge, this is the first report associating K_V_7.5 with a disease.

The physiological relevance of this finding lies in the major impact these channel complexes underlying the M-current have for controlling neuronal excitability (Wang et al., 1998; Schroeder et al., 2000; Cooper and Jan, 2003) and generation of theta oscillations which are involved in memory formation and spatial navigation (Hu et al., 2002; Peters et al., 2005; Wang, 2010). Theta oscillations are not only observed in the hippocampus but also in the surrounding limbic structures as well as in the prefrontal cortex (Wang, 2010). These areas of the brain are involved in memory storage (Morgado-Bernal, 2011), emotional processing (Adolphs, 2010), behavioral monitoring, and valuation of response outcomes (Wang, 2010) which are all aspects of normal brain functioning that appear to be affected in individuals with ASD, ID, or psychiatric disorders and accordingly might contribute to the observed phenotypes of the patients presented here. Since several neurotransmitters, neuromodulators, and pharmacological drugs can influence the properties of M-channels (Cooper and Jan, 2003) it can thus be speculated whether any of these modulators could improve the quality of life for the patients described here and potentially other psychiatric patients.

In conclusion, we here present four unrelated ASD patients with variations in *KCNQ3*. One patient has a truncating *de novo* mutation whereas the other three patients have inherited a c.1720C > T [p.P574S] nucleotide change (rs74582884). One transmitting parent suffers from major depression whereas the other two are phenotypically normal. This SNP was previously reported in patients with rolandic epilepsy, IGE, or benign neonatal convulsions and accordingly, shows varying expressivity and reduced penetrance. The p.P574S change in the K_V_7.3 channel protein significantly reduces currents when co-expressed with K_V_7.5 but not K_V_7.2 or K_V_7.4 in a heterologous expression system. This suggests that specific dysfunction of the K_V_7.3/K_V_7.5 channel may be associated with some forms of ASD, ID, major depression, epilepsy, and possibly other psychiatric disorders and accordingly *KCNQ5* should also be considered a candidate gene for these disorders.

## Conflict of Interest Statement

The authors declare that the research was conducted in the absence of any commercial or financial relationships that could be construed as a potential conflict of interest.
